# Global trends and hotspots in research on acupuncture for stroke: a bibliometric and visualization analysis

**DOI:** 10.1186/s40001-023-01253-w

**Published:** 2023-09-21

**Authors:** Jiale Zhang, Chenyang Ji, Xu Zhai, Shuo Ren, Hongxuan Tong

**Affiliations:** 1 Institute of Basic Theory for Chinese Medicine, China Academy of Chinese Medical Sciences, Beijing, 100700 China; 2 Science and Technology College of Jiangxi, University of Traditional Chinese Medicine, Nanchang, 330004 China; 3 Wangjing Hospital of China Academy of Chinese Medical Sciences, Beijing, 100102 China; 4 Affiliated Hospital of Shandong University of Traditional Chinese Medicine, Jinan, 250011 China

**Keywords:** Acupuncture, Stroke, Bibliometric analysis, Global trends, Brain disease

## Abstract

Acupuncture has been widely used in stroke and post-stroke rehabilitation (PSR), but there is no literature on the bibliometric analysis of acupuncture for stroke. This study aimed to characterize the global publications and analyze the trends of acupuncture for stroke in the past 40 years. We identified 1157 publications from the Web of Science Core Collection. The number of publications grew slowly in the first three decades from 1980 until it started to grow after 2010, with significant growth in 2011–2012 and 2019–2020. China, the USA, and South Korea are the top three countries in this field, and China has formed good internal cooperative relations. Early studies focused on the clinical efficacy of acupuncture for stroke. In the last five years, more emphasis has been placed on the effectiveness of acupuncture in treating sequelae and complications, combined with neuroimaging studies to explore the mechanisms of brain injury repair and neurological recovery. Acupuncture for stroke has a vast research potential, and researchers from different countries/regions and organizations still need to remove academic barriers to enhance communication and collaboration.

**Supplementary Information:**

The online version contains supplementary material available at 10.1186/s40001-023-01253-w.

## Highlights

- For the first time, we analyzed hotspots related to the field of acupuncture for stroke research using a bibliometric approach, focusing on the most critical indicators, including researchers, countries, research institutions, and journals.
- 1157 publications from 60 countries/regions contributed to this research theme. China is the main producer of acupuncture for stroke and has formed a cooperation cluster. It is worth noting that researchers from different countries/regions and organizations still need to remove academic barriers to enhance communication and collaboration.
- Future research trends are focused on providing high-quality clinical evidence, integrating neurological disciplines, exploring new models with multidisciplinary overlap, and exploring the development of artificially intelligent acupuncture robots. The integration of acupuncture with neuroscience and computational science, as well as deep learning and artificial intelligence, are hotspots in acupuncture for stroke.

**Supplementary Information:**

The online version contains supplementary material available at 10.1186/s40001-023-01253-w.

## Introduction

Cerebral stroke is a common cerebrovascular disease, the onset of which is related to impaired blood circulation, vascular rupture, or obstruction of blood flow in cerebral blood vessels, including ischemic and hemorrhagic stroke [1, 2]. Studies [3, 4] showed that the age-standardized incidence rate (ASIR) was the highest for ischemic stroke in East Asia in 2019 and the most significant increase in the ASIR from 1990 to 2019. Globally, ischemic stroke incidence increases, especially in women aged 50 to 69 [5]. In 2019, there were 394 million new stroke cases in China, up by 86.0% compared to 1990 [6]. Contrary to the decreasing trend in developed countries, the incidence of stroke in China increased significantly, and the burden of stroke remains severe and is the primary cause of death [7, 8]. Therefore, the prevention and treatment of cerebral infarction are of great importance.

Acupuncture is widely used in stroke and post-stroke related complications, such as post-stroke impairments in motor function, cognitive function, and mental disorders [9–11]. Acupuncture can significantly improve neurobehavioral function and reduce animal brain infarct volume [12, 13]. Studies on the mechanism of acupuncture for stroke have focused on anti-apoptosis [14], autophagy promoting neural regeneration [15], anti-inflammatory [16], anti-oxidative stress [17], and improving cerebral circulation [18].

Bibliometrics can analyze the contributions of different authors, journals, institutions, and countries to a research topic and discover trends and domain-specific hotspots [19]. Therefore, this study used two widely used bibliometric tools, CiteSpace and VOSviewer, based on the Web of Science core collection (WoSCC) from 1980 to 2022 publications. Our study is the first to describe the current situation and hotspots in acupuncture for stroke in the last 40 years. The objective of this research is threefold: (1) to identify articles and journals with high impact, prolific authors, institutions, and countries/regions with significant contributions; (2) to delineate the central research topics and curreas of interest; and (3) to forecast the future trends of acupuncture for stroke.

## Materials and methods

### Search strategy

**Fig. 1:**
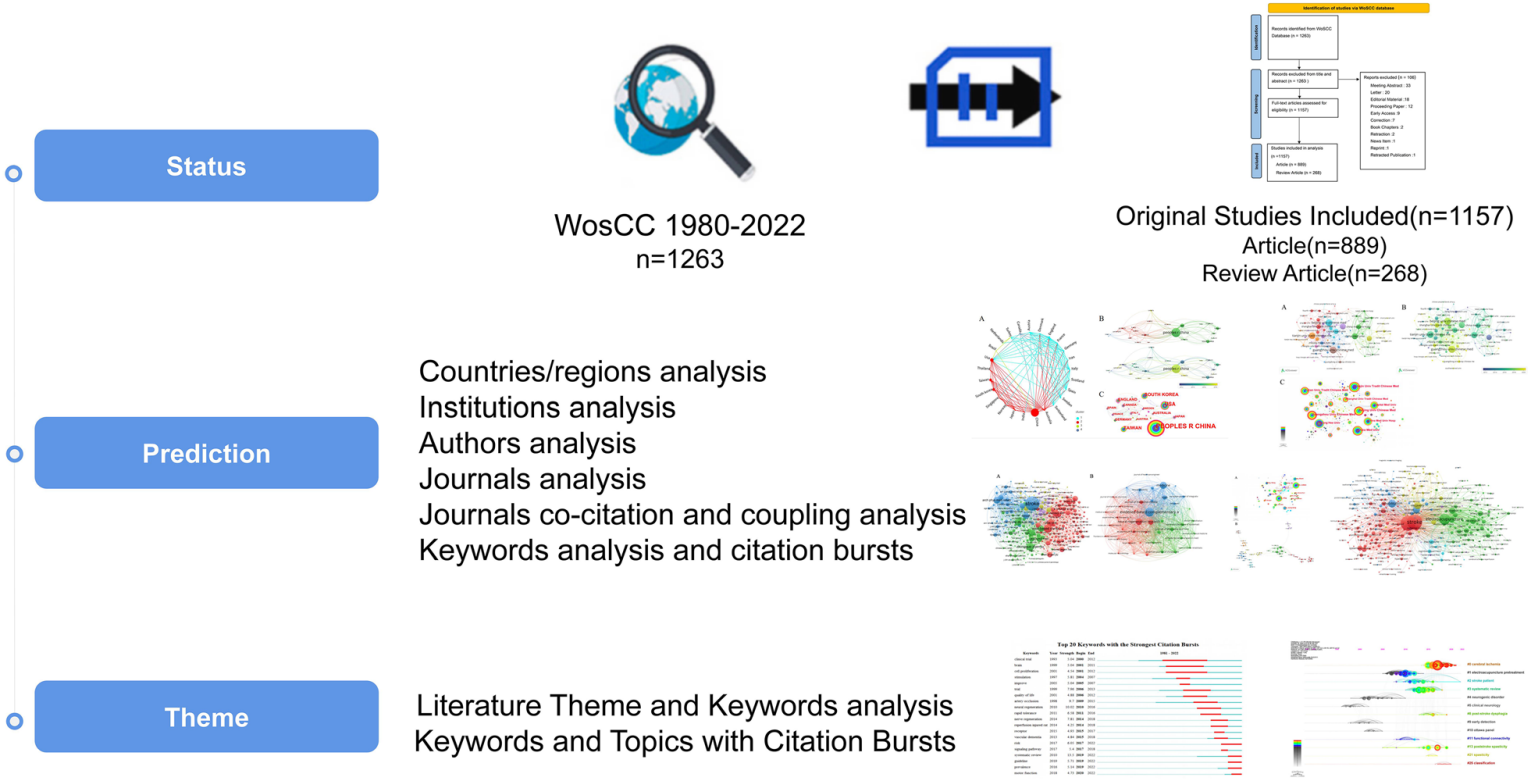
Bibliometric analysis of acupuncture for stroke in the workflow

We conducted a systematic search through the Web of Science Core Collection (WoSCC) [20], which only included the Science Citation Index Expanded (SCI-E) literature included in the library. The retrieval type is TS = (acupuncture or Electroacupuncture or dry needing or acupoint injection)) AND TS = (apoplexy or stroke or cerebral infarction). We excluded meeting abstract, letter, editorial material, proceeding paper, early access, correction, book chapters, retraction, news item, reprint, or retracted publication. Bibliometric analysis of acupuncture for stroke in the workflow is shown in Fig. 1. Two researchers (Jiale Zhang and Chenyang Ji) were assigned to search the database and filter the literature, while a senior researcher (Hongxuan Tong) handled potential controversies and disagreements.

### Data extraction and bibliometric analysis

The included literature was downloaded into Microsoft Excel 2019 for statistical analysis of basic information, including title, keywords, journal, publication date, and other essential information. VOSviewer was adopted to visualize countries/regions, institutions, authorship partnerships, and keyword co-occurrence journal coupling mapping. Among them, based on the countries/regions cooperation graph generated by VOSviewer, we use Scimago Graphica to develop a complex network graph of countries/regions associations, which can better present the cooperation relationship between countries/regions. CiteSpace is used to identify the keywords bursting to show the evolution of the research field. For the concepts mentioned in the study, journal coupling analysis originates from Professor M.M. Kessler in 1963 [21], which states that two journals are coupled if they cite the third journal together. Journal coupling analyzes the scholarly communication between journals and determines the journal’s position and the association between the disciplines. Journal co-citation [22] is a method to explore the association between journals through the external perception of journals. If one or more papers cite two (or more) papers simultaneously, the two are known to constitute a co-citation relationship. Journal coupling analysis the relationship between journals from the perspective of knowledge uptake, while journal co-citation analysis examines the association between journals from the perspective of knowledge output. More detailed instructions on the specific procedures can be found in Additional file 1.

## Result

### Basic analysis of the literature

**Fig. 2:**
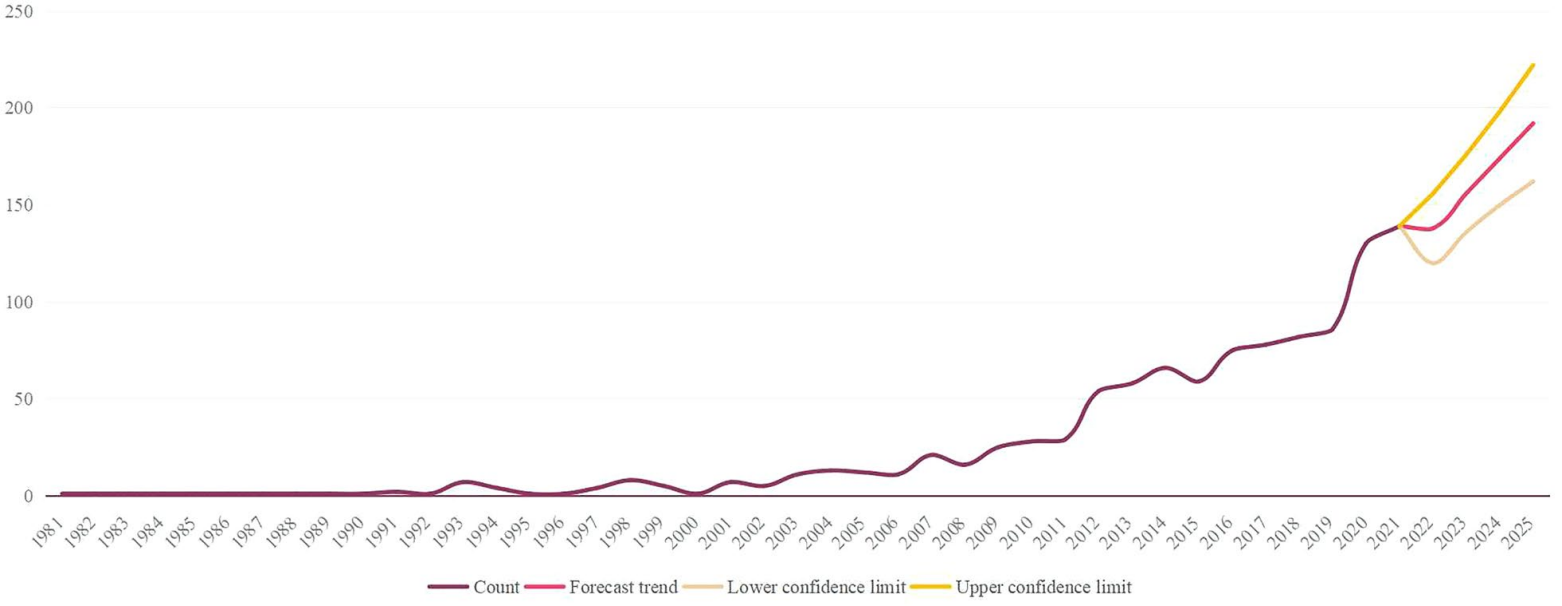
Publication prediction diagram

As shown in Fig. 2, 1157 articles were included, with an overall increasing trend in the number of articles. From 1995 to 2005, the annual output of articles was approximately equal. The literature on acupuncture for stroke increased steadily from 2011 to 2012, indicating the beginning of interest in the treatment. The number of articles increased from 2019 to 2020, reaching 139 outputs by 2021. Based on Microsoft Excel worksheets, we predict that by 2025, the annual literature volume will exceed 200 articles.

### Countries/regions analysis

**Fig. 3:**
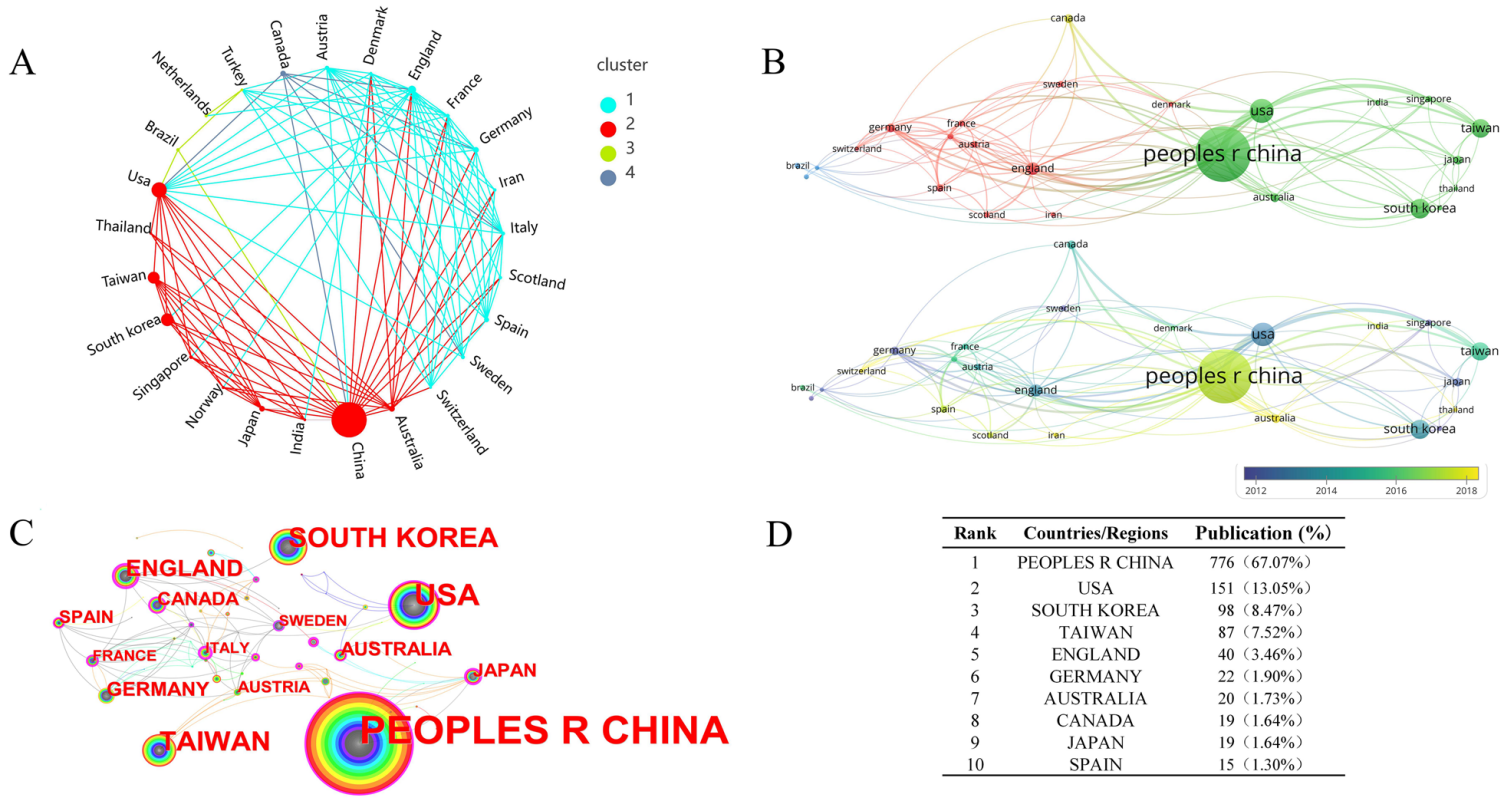
Link density relationships between countries/regions and the top ten countries/regions. **A** The complex network of countries/ regions associations, forming four types of associations; **B **the four types of associations through the form of a map; **C** the intensity of cooperation between countries/ regions; **D** the top ten countries/regions. The most published papers were from the People's Republic of China (*n* = 776), followed by the USA (*n* = 151) and South Korea (*n* = 98)

In total, 60 countries or regions have published papers on treating stroke with acupuncture. Figure 3D shows the top 10 countries and regions with the highest yield. The most published papers were from the People's Republic of China (*n* = 776), followed by the USA (*n* = 151) and South Korea (*n* = 98). Figure 3A–C depicts the connectivity density among East Asian countries, North America, and some European countries. In total, four clusters were formed. Among the different countries, China ranks first in the number of publications. In terms of the timeline, as a typical representative, China started late, despite its size, and after 2015, studies in China began to increase gradually (Fig. 3B).

### Institutions analysis

**Fig. 4:**
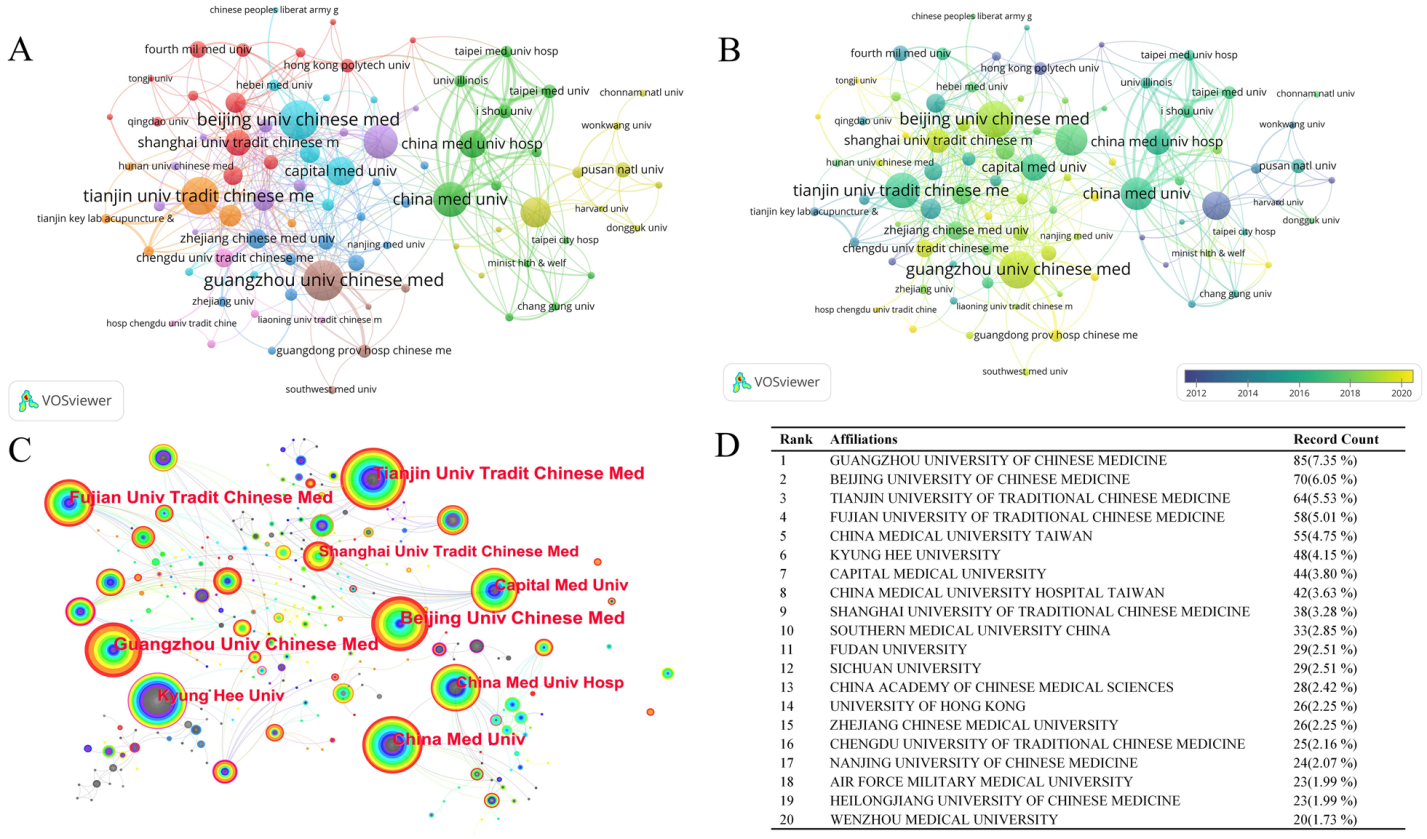
Institutions relationship and collaboration density and the top 20 institutions. **A** The dense and complex lines between various research institutions in China indicate high correlation and close collaboration. **B** The edges with lighter colors (yellow) represent the period after 2016, during which more Chinese institutions were involved in the research. The darker-colored edges represent the earlier period when Chinese institutions were less involved. **C** The color depth represents the chronological order, with brighter colors indicating later periods. It can be observed that research from mainland China appeared later than that from other regions. The dense lines between South Korea and Taiwan represent a close collaboration. D. The top 20 institutions. GZUCM with the most publications of 85 (7.35%) articles, BUCM with 70 (6.05%) articles, and TUTCM with 64 (5.53%) articles

Altogether, there were 1,279 universities and institutes participating in the study (Fig. 4). Figure 4D shows the top 20 institutions. The top five institutions by publication count were GZUCM with 85 (7.35%) articles, BUCM with 70 (6.05%) articles, TUTCM with 64 (5.53%) articles, FJTCM with 58 (5.01%) articles, and CMU with 55 (4.75%) articles. The graph of institutional partnerships and collaboration density shows that China has formed a network of collaborative relationships within the three TCM universities in tandem with comprehensive universities and local universities. In terms of the timeline, the research of China Medical University (Taiwan) and Kyung Hee University in South Korea formed a sizeable cluster centered on research until 2016. After 2016, Chinese research increased and gradually formed a large-scale, established research group.

### Authors analysis

**Fig. 5:**
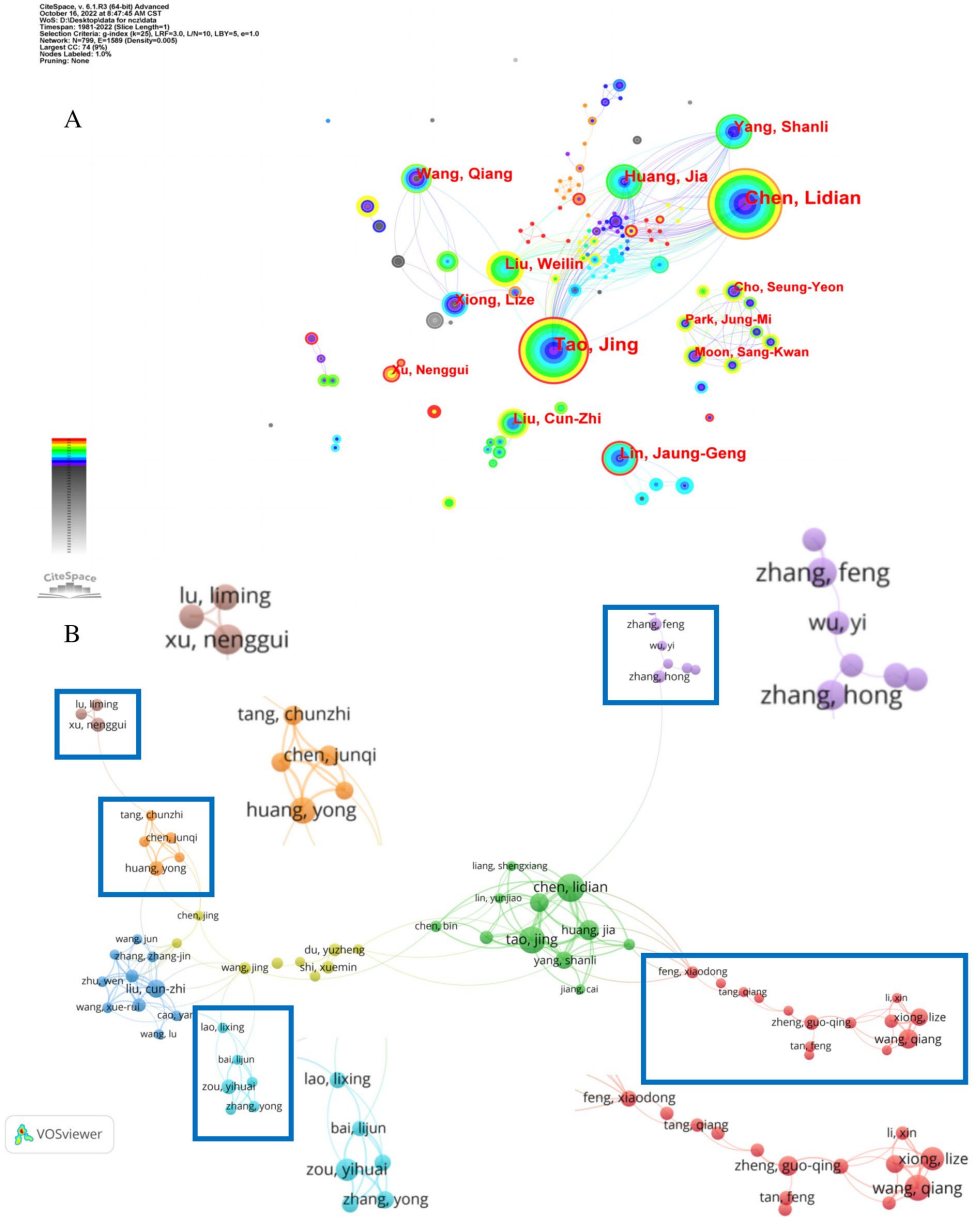
Author collaboration network

A total of 4,293 authors participated in studies on acupuncture for stroke. Chen Lidian was the prolific author with 44 publications, followed closely by Tao Jing (*n* = 41) and Huang Jia (*n* = 25). CiteSpace and VOSviewer visualize the cooperative network between authors. As shown in Fig. 5A, authors within the same country are closely connected, and scientific collaboration is relatively frequent, but the links between different countries and regions remain low.

Figure 5B shows the network diagram based on VOSviewer. The relational network of the author's group collaboration also reflects the size of the study. Overall, five core groups of researchers have been formed in China. Such as Chen Lidian, Liu Cunzhi, Shi Xuemin, Xu Nenggui, and Tang Chunzhi, Wang Qiang. In addition, through the CiteSpace analysis research team, there is also a research group formed in Korea, represented by Seung-Yeon Cho.

### Journals analysis

Out of 1157 publications included, published in 313 different journals. Table 1 shows the top 20 journals. The top three journals are eCAM, Medicine (IF/JCR = 1.817/Q3), Neural Regen Res (IF/JCR = 6.058/Q1), and Integrative & Complementary Medicine accounted for 50% of the top 20 journals, followed by neuroscience or clinical neuroscience, accounting for 30% of the weight.

**Table 1:** Information on the top 20 journals

Rank	Publication titles	Record count	Category	IF/JCR
1	Evidence-Based Complementary and Alternative Medicine	101(8.73%)	Integrative & Complementary Medicine	NA
2	Medicine	69(5.96%)	Medicine, General & Internal	1.817/Q3
3	Neural Regeneration Research	62(5.36%)	Neurosciences	6.058/Q1
4	Acupuncture in Medicine	44(3.80%)	Integrative & Complementary Medicine	1.976/Q3
5	Trials	34(2.94%)	Medicine, Research & Experimental	2.728/Q4
6	Journal of Traditional Chinese Medicine	28(2.42%)	Integrative & Complementary Medicine	2.547/Q3
7	Journal of Alternative and Complementary Medicine	27(2.33%)	Integrative & Complementary Medicine	2.381/Q3
8	BMC Complementary and Alternative Medicine	24(2.07%)	Integrative & Complementary Medicine	4.782/Q1
9	ACUPUNCTURE & ELECTRO-THERAPEUTICS RESEARCH	20(1.73%)	Neurosciences	0.684/Q4
10	Cochrane Database of Systematic Reviews	20(1.73%)	Medicine, General & Internal	12.008/Q1
11	Frontiers in Neurology	20(1.73%)	Integrative & Complementary Medicine	4.086/Q2
12	American Journal of Chinese Medicine	19(1.64%)	Integrative & Complementary Medicine	6.005/Q1
13	Chinese Journal of integrative Medicine	18(1.56%)	Multidisciplinary Sciences	2.626/Q3
14	PLoS One	18(1.56%)	Integrative & Complementary Medicine	3.752/Q2
15	Neural Plasticity	17(1.47%)	Neurosciences	3.144/Q3
16	Stroke	17(1.47%)	Clinical Neurology	10.170/Q1
17	Neurological Research	16(1.38%)	Clinical Neurology	2.529/Q3
18	Complementary Therapies in Medicine	15(1.30%)	Integrative & Complementary Medicine	3.335/Q2
19	Neuroscience Letters	14(1.21%)	Neurosciences	3.197/Q3
20	European Journal of Integrative Medicine	13(1.12%)	Integrative & Complementary Medicine	1.813/Q4

All impact factors are the latest impact factors published by Clarivate in 2021

### Journals co-citation and coupling analysis

**Fig. 6:**
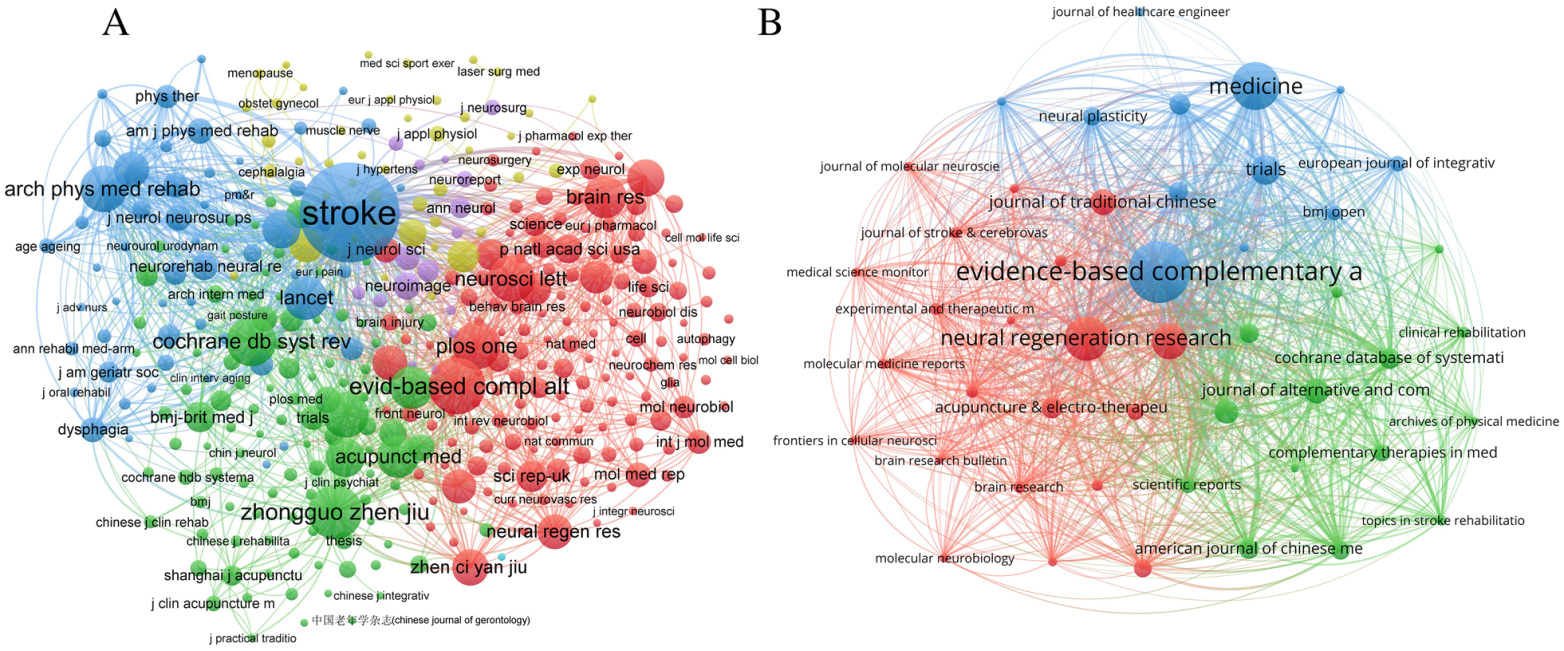
Journal co-citation and journal coupling

In journal co-citation analysis, 7713 articles were extracted by VOSviewer, and we selected 370 papers with citation frequency more significant than 20 to the presentation (Fig. 6A). According to the total citation intensity analysis, the top-ranked journals are “Stroke, Arch Phys Med Rehabil, and eCAM”. In the journal coupling analysis, we set frequency five as the minimum coupling amount, and 48 of 312 journals reached the threshold, but one of the nodes was not associated with other nodes, so only 47 are shown in Fig. 6B. According to the coupling strength, the top three stronger journals are “eCAM, Neural Regen Res, and Acupuncture in Medicine”.

### Literature theme and keywords analysis

**Fig. 7:**
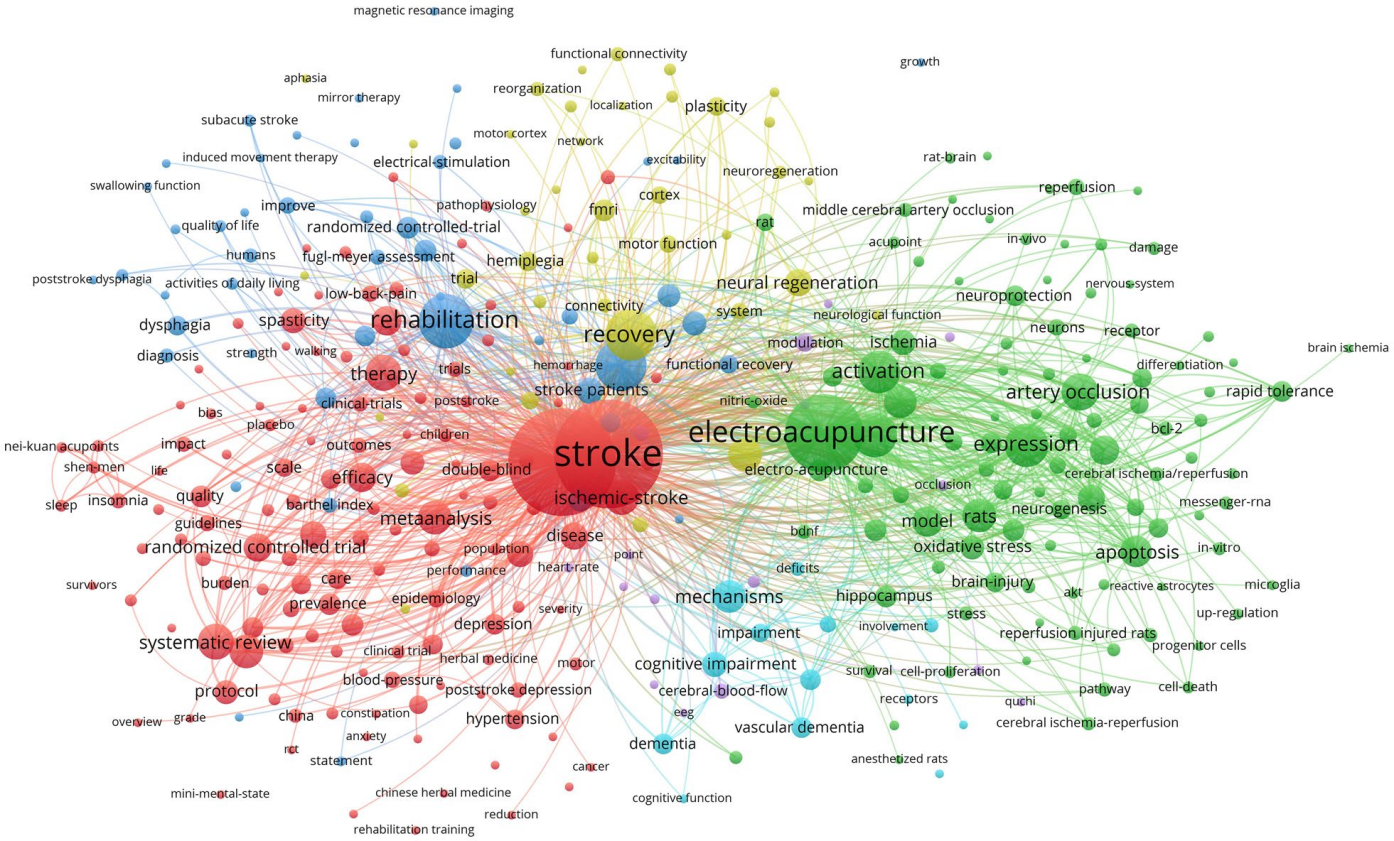
Cluster analysis and cooperative network of keywords

The analysis of the literature reflects the trend of hotspots and research topics. We did a set of cluster analyses and timeline evolution of the literature co-cited. For the analysis of keywords, we used VOSviewer to present a keywords network graph (Fig. 7), with the size of the circles representing the total link strength and the thickness of the lines representing the number of co-occurrences. Several clusters were formed based on the classification of keywords. The red cluster shows keywords related to clinical studies such as randomized controlled trial, double-blind, outcome, scale, guidelines, systematic review, bias, and epidemiology. In addition, above the red cluster, two types of clusters focus on clinical research on PSR, a blue cluster that focuses on quality of life improvement in PSR and a yellow cluster that emphasize the exploration of rehabilitation after stroke through neuroimaging. Below the red cluster is a small category of the light blue cluster, which focuses on stroke for neurological deficits, especially acupuncture interventions for cognitive impairment, vascular dementia, and other disease mechanisms and clinical research. On the right of the red cluster is another green cluster focused on animal experiments research, especially on EA for neurological deficits in animal models. Several relevant subject words, such as neurogenesis, activation, neurons, hippocampus, astrocytes, apoptosis, and rapid tolerance, are associated with exploring the mechanisms and targets of EA against stroke.

### Keywords with the strongest citation bursts

**Fig. 8:**
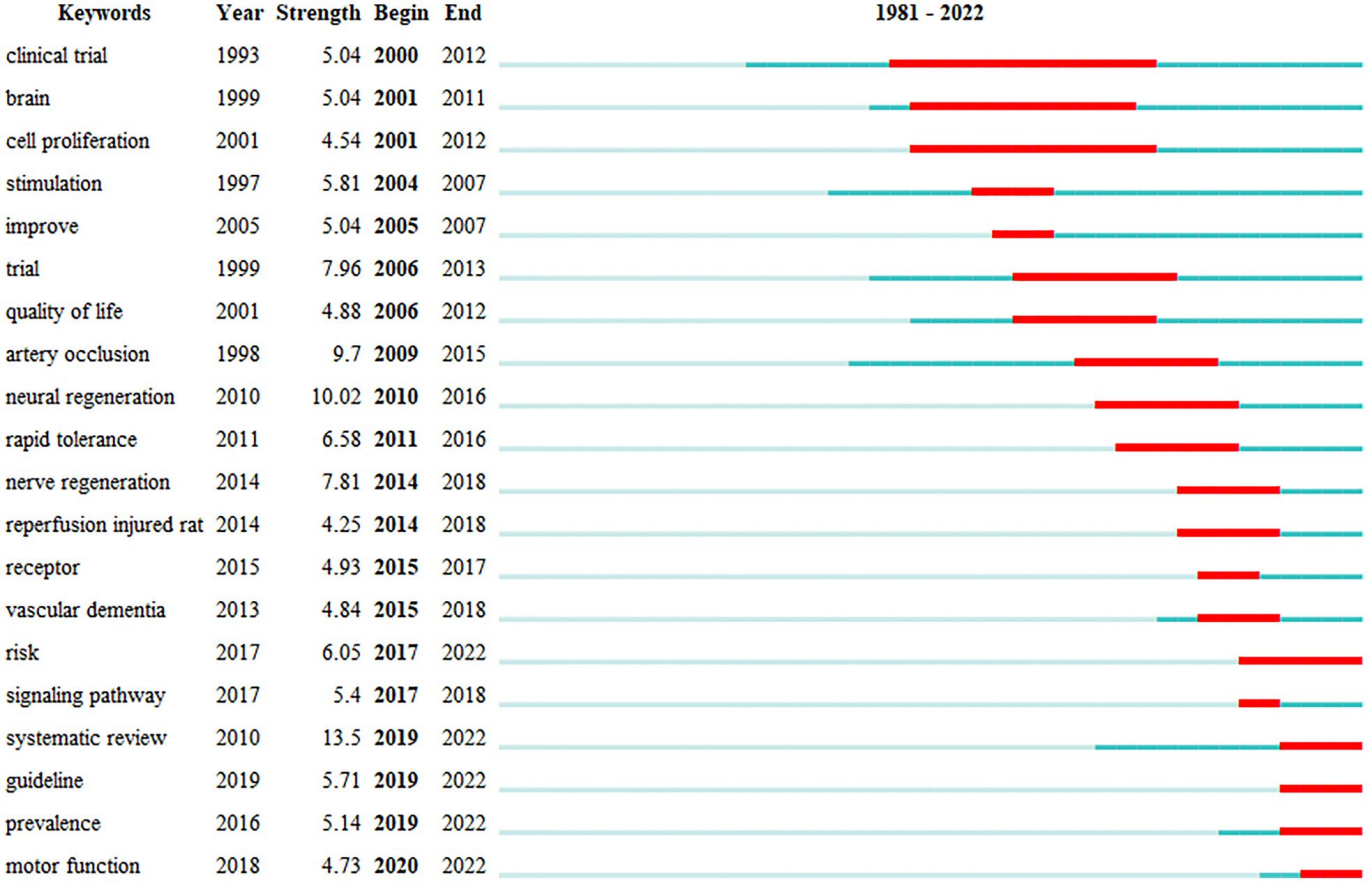
Keywords with the strongest citation bursts

We did a citation burst analysis of keywords (Fig. 8). Clinical research on acupuncture for stroke has been a long-standing hotspot. In the past three years, the focus has been on integrating evidence, including evaluating the reporting quality of randomized controlled trials (RCTs) and the functional recovery after stroke. Table 2 lists the randomized controlled trial (RCT) literature cited for high frequency. In addition, in animal models studies, attention has been paid to acupuncture intervention in neuronal apoptosis in cerebral infarction rats to improve motor ability, learning memory, and neurological function, primarily through signaling pathways such as NF-Κb [23], PI3K/Akt [24], to reduce cerebral ischemic injury and neuroinflammation.

**Table 2:** Top 20 literature of randomized controlled trials

Title	Authors (top five)	Journal title	Impact factor	Year	DOI	Citation
Acupuncture and transcutaneous nerve stimulation in stroke rehabilitation—A randomized, controlled trial	Johansson, BB; Haker, E; von Arbin, M; Britton, M; Langstrom, G; et al	Stroke	10.170/Q1	2001	10.1161/01.STR.32.3.707	118
Clinical trial of electrical acupuncture on hemiplegic stroke patients	Wong, AMK; Su, TY; Tang, FT; Cheng, PT; Liaw, MY	American Journal of Physical Medicine & Rehabilitation	3.412/Q1	1999	10.1097/00002060–199903000-00006	92
A randomized controlled trial on the treatment for acute partial ischemic stroke with acupuncture	HU, HH; CHUNG, C; LIU, TJ; CHEN, RC; CHEN, CH; et al	Neuroepidemiology	5.393/Q1	1993	10.1159/000110308	76
Acupuncture efficacy on ischemic stroke recovery multicenter randomized controlled trial in China	Zhang, Shihong; Wu, Bo; Liu, Ming; Li, Ning; Zeng, Xianrong; et al	Stroke	10.170/Q1	2015	10.1161/STROKEAHA.114.007659	70
Acupuncture for subacute stroke rehabilitation—A sham-controlled, subject- and assessor-blind, randomized trial	Park, J; White, AR; James, MA; Hemsley, AG; Johnson, P; et al	JAMA internal medicine (formerly the Archives of Internal Medicine)^a^	44.409/Q1	2005	10.1001/archinte.165.17.2026	64
Transcutaneous electrical stimulation on acupuncture points improves muscle function in subjects after acute stroke: A randomized controlled trial	Yan, Tiebin; Hui-Chan, Christina W. Y	Journal of Rehabilitation Medicine	3.959/Q1	2009	10.2340/16501977–0325	52
Intradermal acupuncture on Shen-men and Nei-kuan acupoints improves insomnia in stroke patients by reducing the sympathetic nervous activity: A randomized clinical trial	Lee, Seung Yeop; Baek, Yong Hyeon; Park, Seong Uk; Moon, Sang Kwan; Park, Jung Mi; et al	American Journal of Chinese Medicine	6.005/Q1	2009	10.1142/S0192415X09007624	48
Efficacy and safety assessment of acupuncture and nimodipine to treat mild cognitive impairment after cerebral infarction: A randomized controlled trial	Wang, Shuhua; Yang, Hongling; Zhang, Jie; Zhang, Bin; Liu, Tao; et al	BMC Complementary and Alternative Medicine	4.782/Q1	2016	10.1186/s12906-016–1337-0	36
A pilot controlled trial of a combination of dense cranial electroacupuncture stimulation and body acupuncture for post-stroke depression	Man, Sui-Cheung; Hung, Ben H. B.; Ng, Roger M. K.; Yu, Xiao-Chun; Cheung, Hobby; et al	BMC Complementary and Alternative Medicine	4.782/Q1	2014	10.1186/1472–6882-14–255	36
Effects of dry needling on post-stroke spasticity, motor function and stability limits: A randomized clinical trial	Sanchez-Mila, Zacarias; Salom-Moreno, Jaime; Fernandez-de-las-Penas, Cesar	Acupuncture in Medicine	1.976/Q3	2018	10.1136/acupmed-2017–011568	33
Clinical efficacy of acupuncture treatment in combination with rehacom cognitive training for improving cognitive function in stroke: A 2 × 2 factorial design randomized controlled trial	Jiang, Cai; Yang, Shanli; Tao, Jing; Huang, Jia; Li, Yinyan; et al	Journal of the American Medical Directors Association	7.802/Q1	2016	10.1016/j.jamda.2016.07.021	33
Additional effects of acupuncture on early comprehensive rehabilitation in patients with mild to moderate acute ischemic stroke: A multicenter randomized controlled trial	Chen, Lifang; Fang, Jianqiao; Ma, Ruijie; Gu, Xudong; Chen, Lina; et al	BMC Complementary and Alternative Medicine	4.782/Q1	2016	10.1186/s12906-016–1193-y	30
Clinical effects of scalp electrical acupuncture in stroke: A sham-controlled randomized clinical trial	Hsing, Wu Tu; Imamura, Marta; Weaver, Kayleen; Fregni, Felipe; Azevedo Neto, Raymundo S	Journal of Alternative and Complementary Medicine	2.381/Q3	2012	10.1089/acm.2011.0131	28
Bee venom acupuncture point injection for central post stroke pain: A preliminary single-blind randomized controlled trial	Cho, Seung-Yeon; Park, Joo-Young; Jung, Woo-Sang; Moon, Sang-Kwan; Park, Jung-Mi; et al	Complementary Therapies in Medicine	3.335/Q2	2013	10.1016/j.ctim.2013.02.001	25
Therapeutic effect of acupuncture and massage for shoulder-hand syndrome in hemiplegia patients: A clinical two-center randomized controlled trial	Li, Ning; Tian, Fengwei; Wang, Chengwei; Yu, Pengming; Zhou, Xi; et al	Journal of Traditional Chinese Medicine	2.547/Q3	2012	10.1016/S0254-6272(13)60,035–7	20
The effects of acupuncture on cerebral blood flow in post-stroke patients: A randomized controlled trial	Ratmansky, Motti; Levy, Adi; Messinger, Aviv; Birg, Alla; Front, Lilach; et al	Journal of Alternative and Complementary Medicine	2.381/Q3	2016	10.1089/acm.2015.0066	19
Efficacy of integrated rehabilitation techniques of traditional Chinese medicine for ischemic stroke: A randomized controlled trial	Zhang, Yong; Jin, He; Ma, Dayong; Fu, Yuanbo; Xie, Yanming; et al	American Journal of Chinese Medicine	6.005/Q1	2013	10.1142/S0192415X13500651	18
Effectiveness of acupuncture for vascular cognitive impairment no dementia: A randomized controlled trial	Yang, Jing-Wen; Shi, Guang-Xia; Zhang, Shuai; Tu, Jian-Feng; Wang, Li-Qiong; et al	Clinical Rehabilitation	2.884/Q2	2019	10.1177/0269215518819050	17
Traditional Chinese acupuncture for poststroke depression: A single-blind double-simulated randomized controlled trial	Qian, Xiaolu; Zhou, Xuan; You, Yanli; Shu, Shi; Fang, Fanfu; et al	Journal of Alternative and Complementary Medicine	2.381/Q3	2015	10.1089/acm.2015.0084	15
Efficacy and safety of transcutaneous electrical acupoint stimulation to treat muscle spasticity following brain injury: A double-blinded, multicenter, randomized controlled trial	Zhao, Wenli; Wang, Chao; Li, Zhongzheng; Chen, Lei; Li, Jianbo; et al	PLoS One	3.752/Q2	2015	10.1371/journal.pone.0116976	14

^a^JAMA Internal Medicine, formerly known as the Archives of Internal Medicine

All Impact Factor are the latest impact factors published by Clarivate in 2021

## Discussion

In recent years, research surrounding acupuncture for stroke has become increasingly extensive [25–28], especially after 2010, with a faster growth rate. Acupuncture has received increasing attention as a method of complementary and alternative medicine. Although early clinical studies reported negative results [29], subsequent studies have continued to confirm the efficacy of acupuncture for stroke [30] and conduct clinical studies around complications, sequelae, and animal experiments to explore mechanisms of action.

### Main findings

Our study systematically described the status and hot trends of acupuncture for stroke in the past 40 years. Overall, the country with the most published papers was China (*n* = 776), followed by the USA (*n* = 151) and South Korea (*n* = 98). Since acupuncture originated in China with extensive clinical practice and policy support [31, 32], Chinese research institutions have published the most papers. Besides, China Medical University in Taiwan and Kyung Hee University in South Korea have formed regional research centers, respectively. Among the top 10 authors involved in the study, Chen Lidian of FJTCM was the most prolific author. In terms of inclusion, published 313 journals, the top three journals being eCAM, Medicine, and Neural Regen Res. Among the top 20 journals, those classified as Integrative & Complementary Medicine accounted for 50%, followed by neuroscience or clinical neuroscience, occupying 30% of the weight. The results suggest that acupuncture for stroke is mainly published in specialized journals in complementary and alternative medicine and neurological disciplines.

Regarding research themes and hotspots, acupuncture for stroke focuses on clinical and animal experimental studies. In clinical studies, attention is paid to stroke and its related complications, such as insomnia [33], depression [34], hemiplegia [35], etc. In experimental studies, emphasis is placed on the mechanism of action.

We collated two topics based on the visual analysis of VOSviewer and CiteSpace.

### Topic 1: Focus on the integration of evidence and the generation of high-level evidence

Two systematic reviews of “Acupuncture for stroke rehabilitation” were, respectively, reported by the Cochrane Database of Systematic Reviews in 2006 [36] and 2016 [37]. During this decade, the early evidence ensemble suggested a lack of beneficial effects of acupuncture on post-stroke recovery. 2016 updated evidence suggests acupuncture may improve dependency, overall neurological dysfunction, and some specific neurological dysfunction in recovering stroke patients without serious adverse events. Due to the insufficient size of most clinical trials, there is a lack of evidence to support the routine use of the therapy. In addition, a paper published in 2022 on the topic of “Acupuncture for post-stroke with a review of clinical guideline recommendation” [38] mentioned that although clinical practice and treatment guidelines increasingly mention acupuncture as a therapeutic option for post-stroke care, most guidelines indicate a lack of sufficient basis for recommending acupuncture as a treatment option for PSR.

Recent evidence published in 2022 suggests that interactive dynamic scalp acupuncture [39–41] is effective in cognitive function, motor function, and gait of lower limbs after stroke, where acupuncture improves cognitive function but reduces anxiety, depression and ultimately promotes the patient's ability of daily activity. In another study evaluating the role of acupuncture in ischemic stroke rehabilitation [42], after treatment, the acupuncture group had lower NIHSS scores (*P* = 0.017) compared to the traditional training group. The acupuncture was more effective than the traditional training on the Basel Index (*P* = 0.016).

In addition, another category of clinical research focuses on the acupuncture of PSR from the perspective of neuroimaging [43]. Resting-State fMRI before and after acupuncture in stroke patients was found to increase the intrinsically reduced functional connectivity between bilateral primary motor cortices. Thus, further understanding the neuroplasticity mechanism of acupuncture on motor function recovery in stroke is crucial [44].

### Topic 2: Mechanistic studies to explore the action of acupuncture for stroke and its sequelae

In mechanistic studies, acupuncture significantly reduced cerebral infarct volume, improved neurological function, and inhibited neuronal apoptosis [45]. EA modulates endoplasmic reticulum stress in rats with acute ischemic stroke, which significantly increased the mRNA expression level of GRP78, and decreased the expression levels of pro-apoptotic proteins (CHOP / GADD153, p-eIF2 α and caspase 12). The mechanism suggesting that EA protects cells from cerebral ischemia/reperfusion injury neuronal damage may involve the inhibition of endoplasmic reticulum stress [46]. A study [47] on EA intervention in middle cerebral artery occlusion (MCAO) rats from cerebral ischemia–reperfusion injury showed that EA decreased the pro-apoptotic proteins Bax and caspase-3, increased the anti-apoptotic protein Bcl-2, inhibited the transcriptional activity of NF-κB and TRPV1 expression. EA plays an anti-apoptosis role by inhibiting the NF-κB to protect rats from ischemia–reperfusion injury.

As to the neurovascular unit repair, acupuncture plays a vital role by activating the phosphatidylinositol 3-hydroxy kinase/protein kinase B signaling pathway, which has facilitated rehabilitation after cerebral infarction in rats [48].

The combined intervention of mesenchymal stem cell (MSC) transplantation and EA is a neuroprotection strategy for intracerebral hemorrhage (ICH) [49]. The combination of two methods, by relieving cerebral edema and glial scar, promotes neuronal and oligodendrocyte survival, activates mammalian target of rapamycin (mTOR) / 70 kDa ribosomal protein S6 kinase (p70S6K) proteins signaling, and enhances synaptic plasticity [50]. Relevant evidence suggests that the effect of acupuncture in ICH may be related to the modification of microglia polarization via the miR-34a-5p/Krüppel-like factor 4 (Klf4) signaling pathway [51].

On complications and functional recovery, it has been shown that EA is protective against post-stroke depression (PSD). EA reversed depression-like behavior in PSD rats and was better than fluoxetine. The mechanism of action [52] may be related to the activation of the expression of brain-derived neurotrophic factor (BDNF) and its receptor tyrosine kinase receptor B (TrkB) gene. The improvement of depression by EA may be achieved by activating the tissue plasminogen activator (tPA)/BDNF/TrkB pathway [53]. EA attenuates cognitive impairment in stroke rats by regulating endogenous melatonin secretion through synthesizing the aralkylamine N-acetyltransferase gene in the pineal gland. Meanwhile, EA exerts neuroprotective effects and ameliorates cognitive impairment by regulating mitochondrial autophagy-related proteins through melatonin and inhibiting reactive oxygen species induced NLRP3 inflammasome activation [54]. In improving motor function, acupuncture of the MCAO rat model can improve spastic muscle structure partly by enhancing γ-aminobutyric acid and other signaling pathways in the brainstem of spasticity after stroke rats [55]. It was shown that [56] EA at Quchi (LI 11) and Tsusanli (ST 36) enhances motor functional connectivity in brain regions, such as the motor cortex in rats. EA showed high therapeutic microtubule-associated protein 2 expression and motor function recovery after combined rehabilitation training [57]. In the early post-stroke period, EA stimulation can increase the high expression of irisin in the blood and peri-lesion cortex, promote motor function recovery and reduce neuronal death after ischemic stroke in post-ischemic rats [58]. In improving learning and memory, it was shown [59] that nerve growth factor (NGF) entry into the brain promotes learning and memory and inhibits apoptosis of hippocampal neurons in rats. EA enhances the permeability of the blood–brain barrier in the prefrontal cortex and induces NGF uptake by prefrontal neurons, and stimulates NGF into the brain for its therapeutic effects. EA increases intracellular calcium concentration regulated by N-methyl-D-aspartic acid (NMDA) receptor activation. Thus, the hippocampus's 5-HT1A receptor-mediated PKA kinase and NMDA receptors may contribute to improved learning and memory during recovery from EA interventions after ischemic stroke [60]. EA may enhance learning and memory in MCAO-induced cognitive deficit rats by increasing functional connectivity between the retrosplenial cortex and the hippocampus, cingulate gyrus, and midbrain [61].

### Future research trends are focused on the following three points.

They are:

1. Producing high-quality clinical evidence.
2. Integrating neurological disciplines and exploring new models of multidisciplinary overlapping.
3. Exploring the development of PSR acupuncture robots.

The quality of RCT reporting in clinical acupuncture studies still needs to be standardized more. Although the statement of CONSORT 2010 [62] has been published for more than a decade, with the STRICTA 2010 checklist [63] as a specification for reporting acupuncture interventions, it has not yet been widely used in clinical RCT of acupuncture. In the future, clinical studies of acupuncture for stroke will also need to report basic entries according to the study specifications. Since 2015, Chinese scholars have published strong evidence for acupuncture in high-impact journals such as Annals of Internal Medicine [64], JAMA [65], JAMA Internal Medicine [66], and BMJ [67]. Still, high-level evidence for acupuncture for stroke remains to be further studied. In 2020, the NIHR published design methods for optimizing surgically invasive interventions to guide invasive placebo control interventions [68]. These include “Deconstruct (treatment intervention); Identify (critical intervention elements); Take out (critical elements); Think (feasibility and risk of placebo); Optimize (ensure effective blinding)”, a methodological framework (DITTO) to standardize invasive placebo. Studying and learning from the DITTO standardized methodological framework will help to conduct studies on the setting of simulated acupuncture controls and optimize the design of acupuncture placebo interventions for RCT. Evidence-based medicine emphasizes evidence-based scientific decision-making. Regarding evidence translation, some clinical guidelines [69–72] have now incorporated evidence-level recommendations for acupuncture for stroke, such as the Brazilian Practice Guidelines for Stroke Rehabilitation [72], which suggest that acupuncture and EA are recommended to treat post-stroke spasticity. Despite the low level of evidence, there is a need to promote the generation of high-quality evidence so that the most recent evidence can be translated into clinical practice [73] to support clinical decision-making rapidly.

The intersection of acupuncture combined with neuroscience and computational science will be an important development in acupuncture for stroke. Several studies [74–76] have shown that integrating complex central nerves may be the key to acupuncture. Network neuroscience and traditional functional connectivity computing rely on a node-centric network model. Recently the concept of ‘edge-centric’ [77] has been proposed, focusing on analyzing the relationships between the edges constituted by brain regions. The combined use of several techniques, such as brain waves combined with functional magnetic resonance imaging or machine learning techniques for predicting the efficacy of acupuncture, can be carried out in the future. Chinese research teams have recently proposed the concept of computational acupuncture [78], with features such as histology, mathematical modeling, and high-performance computing. Through data mining and knowledge discovery, hypotheses are formed by extracting the hidden patterns behind a large amount of acupuncture data. After hypotheses are formed, mathematical models are built, and computer simulations are used to test the hypotheses and provide predicted results for further in vivo and in vitro experimental studies. This original concept organically links "Discovery Science" and "Hypothesis-Driven Science”, which may be helpful for future research.

Acupuncture combined with deep learning and artificial intelligence rehabilitation robots is also the hotspot of future research [79]. The existing clinical research on acupuncture revolves around acupuncture methods such as EA and dry needling and focuses on the rehabilitation of stroke sequelae, mainly by manual manipulation and supplemented by tools. With the COVID-19 pandemic not yet fully over, combining material technology with engineering to explore the development of acupuncture robots of artificial intelligence. It may contribute to rehabilitating neurological and motor functions in patients with post-stroke sequelae. Currently, there are upper limb exoskeleton robots [80, 81], including the design and development of remote rehabilitation robots [82]. The acupuncture robots will be equipped with various sensors, such as mechanical and electrical sensors, to reduce the pain of needle injections. Furthermore, the design included a study protocol [83, 84] for acupoint positioning, mechanical stimulation, and detection of deqi, for which strategies have been developed.

## Limitation

First, our study was based on a WoSCC search of papers on acupuncture for stroke. Although WoSCC is the most authoritative database, other critical databases are also widely accepted by researchers. Still, the core libraries we selected, especially SCI-E, represent the current status and trends. Second, the citation count of review and original research has the advantage of ensuring the accuracy and quality of the study and can extract high-quality research content. However, including articles in the quantitative analysis may raise concerns about validity due to potential bias. Despite this, our study provides a balanced and informative overview of the evidence base for acupuncture treatment for stroke. In the presentation of the figure, we emphasized the prominent and significantly changed nodes in the results. However, this does not imply that other nodes are unimportant. Furthermore, regarding Table 1, as only the top 20 targets were selected for analysis without considering the remaining research targets, the total percentage does not exceed 100%. Therefore, we must acknowledge this potential limitation to avoid any potential controversy. Last, it is worth noting that the first and corresponding authors in research collaborations are crucial, and simple counts of co-authorships may not fully reflect their contributions. However, we focused on analyzing author collaborations based on co-authorship networks to identify scientific collaboration patterns. Future studies may consider new methods, such as descriptive, diagnostic, predictive, and prescriptive analytics [85, 86].

## Conclusion

This bibliometric analysis summarizes information on countries, institutions, authors, and journals and maps the knowledge network of acupuncture for stroke. Further directions can be considered: (1) focus on the latest evidence-based medical research methods and use the new techniques to guide clinical practice. (2) Strengthen cooperation between researchers, and institutions in acupuncture for stroke, especially between China and other countries, to integrate acupuncture in the brain and neurological disciplines. (3) Develop more acupuncture rehabilitation equipment and standardized acupuncture rehabilitation tools.

### Supplementary Information

**Additional file 1. **The emergence of bibliometrics: a workflow of six steps.

## Data Availability

Data are available on request from the authors.

## Abbreviations

PSR: Post-stroke rehabilitation
EA: Electroacupuncture
ASIR: Age-standardized incidence rate
WoSCC: Web of Science core collection
SCI-E: Science Citation Index Expanded
GZUCM: Guangzhou University of Chinese Medicine
BUCM: Beijing University of Chinese Medicine
TUTCM: Tianjin University of Traditional Chinese Medicine
FJTMC: Fujian University of Traditional Chinese Medicine
CMU: China Medical University (Taiwan)
eCAM: Evidence-based complementary and alternative medicine
Neural Regen Res: Neural regeneration research
Arch Phys Med Rehabil: Archives of Physical Medicine and Rehabilitation
RCTs: Randomized controlled trials
RCT: Randomized controlled trial
NIHSS: National Institutes of Health Stroke Scale
fMRI: Functional magnetic resonance imaging
MCAO: Middle cerebral artery occlusion
PSD: Post-stroke depression
BDNF: Brain-derived neurotrophic factor
