# Supplementary material for: Global trends and hotspots in research on acupuncture for stroke: a bibliometric and visualization analysis
Source: Eur J Med Res. 2023 Sep 21;28:359. doi: 10.1186/s40001-023-01253-w (PMC10512511; doi:10.1186/s40001-023-01253-w)
Supplement: Supplementary file 1 — Additional file 1. The emergence of bibliometrics: a workflow of six steps. [file 40001_2023_1253_MOESM1_ESM.docx]

**Additional file 1**

**The emergence of bibliometrics: a workflow of six steps**

We have outlined a six-step workflow for the emergence of bibliometrics, which includes: Literature screening, Inclusion and exclusion of literature, Literature import, Software operation, Generation of visualizations, Interpretation of visualization.

1. **Literature screening**

Relevant literature was identified by conducting a comprehensive search of prominent academic databases, Web of Science, based on the research topic. The search strategy was designed to retrieve appropriate publications for inclusion in the study.

1. **Inclusion and exclusion of literature**

After completing the search, the retrieved records were exported into reference management software (EndNote) or further screened directly within the databases to exclude publications lacking important information such as subject terms, authorship, and institutional affiliation. This process was undertaken to determine the final set of literature to be included in the study.

1. **Literature import**

**Citespace:**

1. Before using this software, it is necessary to establish a database for the study, which includes the research span (time) and slices (time nodes, typically 1-2 years) of the included literature.

2. The database of the literature source is determined; for instance, in the case of Web of Science (WOS), WOS is selected during the import process. The software automatically decodes and removes duplicates to determine the final number of literature included in the study. (It is worth noting that any database should retain literature data as comprehensively as possible when exporting literature. Therefore, WOS should select "full record with cited references.”)

**VosViewer：**

1. After downloading the data, select "Create" - "Create a map based on bibliographic data" - "Read data from bibliographic database files" and then import the downloaded text (please note the quantity of text, it is recommended to import all at once if the quantity is large).

2. Select "Type of analysis", "Unit of analysis" and the maximum number of authors per document for clustering display based on actual needs.

3. After completing the above operations, a specific literature preview and clustering scale adjustment interface will be operated according to the actual situation.

1. **Software operation**

**Citespace:**

1. After selecting the pre-established database, the data can be analyzed directly according to the needs. (This process mainly includes time slicing/analysis rules/analysis object restrictions)

2. After the settings are completed, the literature data analysis begins.

**VosViewer:**

1. Open the imported literature data in VOSviewer and enter the "Data source" interface.

2. different analysis modes can be selected in the data sources interface, such as co-occurrence analysis, citation analysis, and topic evolution analysis. After selecting the corresponding mode, analysis parameters such as thresholds, time ranges, and analysis units can be set.

3. Click the "Analyze" button, and VOSviewer will automatically perform the analysis and generate corresponding visualizations.

4. In the visualization, adjustments, and optimizations can be made as needed, such as adjusting node size, color, and link thickness. A specific node can also be selected to view detailed information, such as the title, author, and abstract.

5. Mouse interactions and keyboard shortcuts can select, drag, zoom in, and zoom out nodes for better data observation and analysis.

6. It should be noted that the specific steps for using VOSviewer may vary depending on the analysis mode and data source format. It is recommended to carefully read the relevant instructions and tutorials before using the software.

1. **Generation of visualization**

After conducting a literature analysis, the user can adjust node size, color templates, line styles (curved or straight), and clustering size in the settings interface. Additionally, users can directly drag nodes to create their desired image.

Citespace provides a clustering analysis feature through the "Topic Evolution" function. Users should open the previously analyzed literature data in Citespace to perform clustering analysis and enter the "co-occurrence network" or "citation relationship" view. Users should then select the "Analysis" menu and choose "Topic Evolution" to open the topic evolution window. In this window, users can adjust clustering parameters such as clustering algorithm, number of clusters, and clustering threshold to obtain more suitable clustering results. After clicking the "Start Evolution" button, Citespace will automatically perform clustering analysis and generate a topic evolution graph. Each node in the topic evolution graph represents a topic, with its size and color indicating the importance and characteristics of the topic. The connections between nodes represent the relationships between topics, with the thickness and color indicating the relationships' strength and type. Users can adjust the size and position of the topic evolution graph by dragging and zooming in/out to facilitate data analysis. By selecting a node in the topic evolution graph, users can view detailed information about the topic, such as its keywords and literature list, which provides a more comprehensive understanding of the topic's development trends and research hotspots. It should be noted that clustering analysis requires selecting nodes with similar features, such as literature nodes with similar topics or keywords. When setting clustering parameters, users should adjust them based on specific analysis needs and data characteristics to obtain more meaningful and interpretable clustering results. Additionally, Citespace provides other clustering analysis functions, such as keyword clustering and author clustering, which can be selected based on different analysis needs.

**Keyword burst graphs.** Citespace offers a "Hotspot Analysis" feature to generate keyword burst graphs. Users should open the previously analyzed literature data in Citespace to utilize this feature and enter the "co-occurrence network" or "citation relationship" view. From here, users can select the nodes for keyword burst analysis by dragging the mouse or using keyboard shortcuts. Next, users should select the "Analysis" menu and choose "Hotspot Analysis" to open the hotspot analysis window. In this window, users can adjust analysis parameters such as time range, number of keywords, and window size to obtain more appropriate analysis results. These parameters can be adjusted as necessary to optimize the analysis. Finally, clicking the "Start Analysis" button automatically generates the keyword burst graph.

1. **Interpretation of visualization**

**Cluster graph.** The clustering graph (topic evolution) in Citespace represents each node as a topic, with its size and color indicating the importance and characteristics of the topic. The connections between nodes represent the relationships between topics, with the thickness and color indicating the relationships' strength and type. Users can adjust the size and position of the topic evolution graph by dragging and zooming in/out for better data analysis. By selecting a node in the topic evolution graph, users can view detailed information about the topic, such as its keywords and literature list, providing a more comprehensive understanding of the topic's development trends and research hotspots. It should be noted that clustering analysis requires selecting nodes with similar features, such as literature nodes with similar topics or keywords. When setting clustering parameters, users should adjust them based on specific analysis needs and data characteristics to obtain more meaningful and interpretable clustering results. Additionally, Citespace provides other clustering analysis functions, such as keyword clustering and author clustering, which can be selected based on different analysis needs. These clustering analysis functions can provide additional insights into the relationships between topics and authors. Selecting the appropriate clustering analysis function based on the specific research question or objective is important. Overall, the clustering graph in Citespace is a powerful tool for visualizing and analyzing the relationships between topics in a body of literature and can help researchers identify research trends and hotspots in their field.

**Keyword burst analysis.** Citespace automatically performs keyword burst analysis and generates a keyword burst graph. Each node in the keyword burst graph represents a keyword, with its size and color indicating the importance and characteristics of the keyword. The connections between nodes represent the relationships between keywords, with the thickness and color indicating the relationships' strength and type. Users can adjust the size and position of the keyword burst graph by dragging and zooming in/out for better data analysis. The keyword burst graph in Citespace is a valuable tool for identifying important keywords and their relationships in a body of literature. It can help researchers gain insights into the development of research topics over time.

**Notes:**

Pro. Chaomei Chen provides use of the software in Citespace's website download.

<http://cluster.cis.drexel.edu/~cchen/citespace/download/>

How to Use CiteSpace

https://leanpub.com/howtousecitespace

This is an eBook on how to use CiteSpace, especially for beginners and users at the intermediate level. However, it is certainly valuable for users across all levels of proficiency with CiteSpace. The eBook will be updated to reflect new releases of the software.

Mac

You may install Java on your Mac with Homebrew, a package manager for macOS.

To install Homebrew, enter the following in a terminal window:

/usr/bin/ruby -e "$(curl -fsSL https://raw.githubusercontent.com/Homebrew/install/master/install)"

To install Java with Homebrew:

brew cask install java

CiteSpace is a self-signed Java application. You may need to adjust your Java security settings on your computer to allow self-signed applications such as CiteSpace to run on your computer. Here is a step-by-step guide of how to do it on Mac.

A shell script for launching CiteSpace is included in the CiteSpace package: StartCiteSpace_Mac.sh. At the command prompt in a terminal on your Mac, type: sh StartCiteSpace_Mac.sh, then follow the prompt.

In Citespace, the burst strength of a keyword is calculated using the following formula:

Burst strength = (Keyword frequency - Average frequency) / Standard deviation

Here, the average frequency refers to the average number of times all keywords appear in the literature, and the standard deviation refers to the standard deviation of the frequency of all keywords. This formula aims to determine whether the frequency of a keyword appearing in a certain time period is higher than the average level, and whether this high-frequency occurrence is significant. If the burst strength value is greater than 1.0, it indicates that the frequency of the keyword appearing in that time period is higher than the average level, and this high frequency occurrence is significant. If the burst strength value is less than 1.0, it indicates that the frequency of the keyword appearing in that time period is lower than the average level, and this low frequency occurrence is not significant. It should be noted that the calculation of burst strength is based on the frequency of keywords appearing within a certain time period, so the selection of the time period impacts on the calculation results. Additionally, the calculation of burst strength is also limited by the data sample, so when using Citespace for keyword burst analysis, it is important to carefully select the time period and data sample to obtain accurate results. Furthermore, the calculation of burst strength should be analyzed in conjunction with other indicators, such as keyword frequency and co-occurrence, to understand the evolution trend and influence of the keywords fully.

**Reference link:**

As the method for learning this software is through Chinese links, this is provided for learners in a Chinese language environment.

<https://zhuanlan.zhihu.com/p/373065485>

<https://blog.csdn.net/weixin_58566962/article/details/127656104>

<http://cluster.ischool.drexel.edu/~cchen/citespace/manual/CiteSpaceChinese.pdf>

Through our investigation, we discovered videos of Professor Chen Chaomei on YouTube. However, we cannot access and view these relevant videos for learning purposes due to network restrictions. Therefore, we have found the blog to access the information.

<https://blog.sciencenet.cn/home.php?mod=space&uid=496649&do=blog&view=me&from=space>
